# Is the global health community prepared for future pandemics? A need for solidarity, resources and strong governance

**DOI:** 10.15252/emmm.201606337

**Published:** 2016-04-21

**Authors:** Tikki Pang

**Affiliations:** ^1^ Lee Kuan Yew School of Public PolicyNational University of SingaporeSingaporeSingapore

**Keywords:** Microbiology, Virology & Host Pathogen Interaction

## Abstract

In the wake of recent outbreaks of Zika, Ebola and the MERS‐CoV viruses, many are asking: how prepared is the global public health community to deal with future emerging pandemics? Collective action at national, regional and global levels is the best way forward.

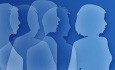

In the wake of recent outbreaks of Zika, Ebola and the MERS‐CoV viruses, and with trust in global institutions at an all‐time low, many are asking: how prepared is the global public health community to deal with future emerging pandemics? A recent report by the Commission on the Global Health Risk Framework, convened by the US National Academy of Medicine, stated that “we are underinvested and underprepared” (GHRF Commission, 2016). What needs to be done? Our experience so far shows that collective action at national, regional and global levels, backed by strong political will and sufficient resources, is the best way to enhance pandemic preparedness and deal with what is not just a health, but a much wider global security issue.

First, any effective global response to pandemics will be only as effective as national preparedness, as “the best way to prevent the global spread of diseases is to detect and contain them while they are still local” (Rodier *et al*, 2007). The Ebola crisis in West Africa demonstrated the dire consequences of fragile health systems unprepared to deal with a massive epidemic. National preparedness is based on the capacities for surveillance, rapid diagnosis, case management, a trained health workforce and surge capacity within the health infrastructure to deal with large numbers of affected persons. WHO's International Health Regulations (IHR), which covers surveillance, monitoring, containment and core capacity building, serves as a guide for countries to strengthen their national health infrastructure and pandemic preparedness. Within a larger context, the strengthening of health systems through provision of universal health care is necessary to achieve the Sustainable Development Goals (SDGs) (Touraine *et al*, 2014), and pandemic preparedness must be seen as a part of the overall health infrastructure in a holistic and integrated manner.

Unfortunately, many developing countries are not sufficiently prepared to deal with an emerging pandemic owing to limited resources, competing priorities and lack of political commitment. An ongoing concern, for example, is the weak and patchy implementation of the IHR, which would better prepare these countries for dealing with pandemics (Lancet, 2014). To underline this urgent need to upgrade public health infrastructure and capabilities of low‐ and middle‐income countries, a recent report proposed spending US$3.4 billion/year to improve global resources for pandemic preparedness and responses (GHRF Commission, 2016).

Second, better regional preparedness will facilitate early warning of potential pandemics and improve international coordination of collective actions for its containment. Effective regional cooperation requires a platform for dialogue and action based on solidarity, trust and goodwill, and, most importantly, a commitment to sharing information rapidly and openly. In South‐East Asia, for example, ASEAN (Association of Southeast Asian Nations) provides a high‐level political and strategic platform for coordinated action, which is supported by regional surveillance initiatives, such as the Mekong Basin Disease Surveillance network. Elsewhere, plans by the European CDC and the African Union to create an African CDC are similarly important initiatives.

There are other strong arguments in favour of regional responses over a more slowly evolving global response. Countries in the region are in closer contact with each other, understand each other and are in a better position to quickly render assistance in the spirit of “helping neighbours”. While efficient regional responses remain a laudable goal, the approach has been dogged by the fairly low profile of health issues in most developing countries, which has, in turn, resulted in a lack of commitment, expertise and resources.

Third, in the face of a potential global spread of a pandemic, global responses must continue to play a central role. WHO's GOARN (Global Outbreak and Alert Response Network) remains at the centre of global coordination efforts but, of course, relies on efficient, accurate and rapid reporting from affected countries. At a higher policy level, WHO continues to be the major international public health agency with a mandate to declare a Public Health Emergency of International Concern (PHEIC), which it has done three times in relation to pandemic influenza, the resurgence of polio and Ebola. Besides alerting the world to a potential pandemic, a PHEIC declaration raises global awareness, facilitates coordinated action and, importantly, helps to mobilize resources to mitigate the impacts of a pandemic. At the front lines, global responses by international NGO's such as Doctors Without Borders (MSF) and the International Red Cross have also been critical in dealing with rapidly deteriorating situations, such as with Ebola in West Africa.

But, as with national and regional preparedness, the recent pandemics have highlighted shortcomings of the global responses. There has been strong criticism, for example, of WHO's inadequate response to the Ebola crisis. An independent expert panel concluded that the organization was slow in recognizing the severity of the situation and did not issue a PHEIC until 5–6 months after the problem began to emerge as a serious threat (Maurice, 2015). The panel also determined that WHO did not have the capacity “to deliver a full emergency public health response” against a severe epidemic outbreak.

However, there are things what WHO can and cannot do. The WHO is not an organization, which can, in 48–72 h, mobilize 100 doctors, 100 nurses and 100 tons of equipment and then transport them to crisis hotspots around the world. Despite the fact that one of its core functions is “to provide technical support to Member States”, it is not an emergency response organization and it is not equipped to do so. What it can, and perhaps should have done, was to be a information clearing house to more rapidly alert the world of the impending emergency. The delay was partly attributed to WHO's organizational structure and delays in information flows between its regional offices and its headquarters in Geneva (Maurice, 2015). MSF, on the other hand, responded heroically on the front lines but was soon overwhelmed by the sheer magnitude of the Ebola outbreak. WHO has borne the brunt of the criticisms, but questions have also been raised about the role of other entities during the Ebola crisis, including the World Bank, the governments of the affected countries, NGOs and other humanitarian groups, and the African Union. In addition, bilateral government responses from the militaries and agencies of the USA, the UK and France were instrumental in helping to deal with the Ebola crisis, but such responses have important diplomatic and political repercussions, which require more analysis and dialogue.

The expert panel report recommended many remedial actions for WHO to be better prepared in the future, such as forming a new Centre for Emergency Preparedness and Response (Maurice, 2015). Beyond these recommendations, another key question that is being asked by many is “what new structures beyond WHO and MSF might be needed to efficiently and effectively address emerging pandemics at the global level?”

Before addressing the issue of creating new global structures, we should clarify some of the criteria and requirements needed for a more effective global public health response to future pandemics. Four are proposed: (i) reforming, consolidating and strengthening key WHO functions; (ii) providing significant and sustainable resources to rapidly responding to outbreaks: suggestions have been made, for example, for a contingency fund of US$100 million to be established at WHO and a much larger Pandemic Emergency Facility to be set up between WHO and the World Bank; (iii) high‐level political commitment and mechanisms to ensure rapid and coordinated global action, perhaps through the imprimatur of the UN Security Council which declared a resolution on Ebola (Gostin & Friedman, 2014); and (iv) the need for a more multi‐sectoral approach. For instance, in lieu of the fact that many emerging diseases have animal origins, organizations involved in animal health should also participate, through a “One Health” approach, in joint monitoring and surveillance efforts (McCloskey *et al*, 2014).

Are such new structures really needed to fulfil these roles efficiently and globally? While it may be attractive in the current atmosphere of disappointment with global institutions to propose a new “Global Fund for Health” (Ooms & Hammonds, 2014), this decision should not be taken lightly. There are already many entities and the creation of new structures has huge implications with regard to the risk of fragmentation of efforts, governance, resources and political issues, which extend well beyond health. Prudence dictates that strengthening existing structures and mechanisms, based on the lessons learned from recent pandemics, would be a better strategy. To give the needed effort the necessary political boost, it has been suggested that a high‐level summit meeting should be convened, ideally by a respected third party outside of the United Nations, which will allow all interested parties to objectively compare their diagnoses of what is needed, and suggest solutions for enhancing preparedness (Garrett, 2015). Such an opportunity may occur in May 2016 during the G‐7 Summit at Ise‐Shima in Japan where outbreak preparedness is likely to be on the agenda as part of the broader theme of human security.

In conclusion, global preparedness for future pandemics must be considered at three closely inter‐connected levels: national, regional and global. Strong national public health infrastructures, effective coordination, solidarity, goodwill, trust, sufficient resources, and a strong emphasis on decisive, collective and rapid action are the foundations for a better global response system to deal with future pandemics. It will also be important to manage the tensions that exist between national sovereignty and the importance of international collective action. The time to act is now or “there is genuine danger that financial commitments from the G‐7 nations, disease surveillance promises made by 194 nations, and essential improvements needed in the global governance of outbreaks will all simply fade off into the sunset of forgotten urgency” (Garrett, 2015).
